# Patient Safety in Medication Nomenclature: Orthographic and Semantic Properties of International Nonproprietary Names

**DOI:** 10.1371/journal.pone.0145431

**Published:** 2015-12-23

**Authors:** Rachel Bryan, Jeffrey K. Aronson, Pius ten Hacken, Alison Williams, Sue Jordan

**Affiliations:** 1 Swansea University, Swansea, Wales, United Kingdom; 2 Nuffield Department of Primary Care Health Sciences, University of Oxford, Oxford, United Kingdom; 3 Institute for Translation Studies, University of Innsbruck, Innsbruck, Austria; Penn State College of Medicine, UNITED STATES

## Abstract

**Background:**

Confusion between look-alike and sound-alike (LASA) medication names (such as mercaptamine and mercaptopurine) accounts for up to one in four medication errors, threatening patient safety. Error reduction strategies include computerized physician order entry interventions, and ‘Tall Man’ lettering. The purpose of this study is to explore the medication name designation process, to elucidate properties that may prime the risk of confusion.

**Methods and Findings:**

We analysed the formal and semantic properties of 7,987 International Non-proprietary Names (INNs), in relation to naming guidelines of the World Health Organization (WHO) INN programme, and have identified potential for errors. We explored: their linguistic properties, the underlying taxonomy of stems to indicate pharmacological interrelationships, and similarities between INNs. We used Microsoft Excel for analysis, including calculation of Levenshtein edit distance (LED). Compliance with WHO naming guidelines was inconsistent. Since the 1970s there has been a trend towards compliance in formal properties, such as word length, but longer names published in the 1950s and 1960s are still in use. The stems used to show pharmacological interrelationships are not spelled consistently and the guidelines do not impose an unequivocal order on them, making the meanings of INNs difficult to understand. Pairs of INNs sharing a stem (appropriately or not) often have high levels of similarity (<5 LED), and thus have greater potential for confusion.

**Conclusions:**

We have revealed a tension between WHO guidelines stipulating use of stems to denote meaning, and the aim of reducing similarities in nomenclature. To mitigate this tension and reduce the risk of confusion, the stem system should be made clear and well ordered, so as to avoid compounding the risk of confusion at the clinical level. The interplay between the different WHO INN naming principles should be further examined, to better understand their implications for the problem of LASA errors.

## Background

Medication errors make up a high proportion of all events related to patient safety [1,2], and are particularly common in intensive care, paediatrics/neonatology, care of the elderly, anaesthetics, and obstetrics [2,3]. Some medication errors will result in overdose, adverse drug reactions, or under-treatment, and cause serious harm to patients [4–6]. As more medications enter the market, with greater variation in routes of administration, this problem is becoming increasingly complex [7].

Errors can occur when medications have similar-looking or similar-sounding names; these are called look-alike, sound-alike (LASA) errors. LASA errors are estimated to account for around one in every four medication errors in the USA [8], and they can occur during prescribing, transcribing, dispensing, and administration (examples in Table 1). Studies of United States Adopted Names (USANs), many of which take the form of International Nonproprietary Names (INNs), have shown that the prescribing frequency of certain medications may prime the risk of LASA errors, and certain pre-approval strategies have been recommended, such as computerized searches, expert judgement, and psycholinguistic testing [9]. Most literature on LASA errors, involving confusion between both brand and generic names (brand-brand, generic-brand, and generic-generic), deals with mitigation strategies and regulatory obligations, such as ‘Tall Man’ lettering on packaging to highlight distinguishing characters (for example, lamoTRIGine/lamiVUDine) and technological solutions, such as alerts built into prescription software and automated reporting systems [4,8,10–12].

**Table 1:** Examples of LASA errors.

Reference	Medications involved (International Nonproprietary Name)	Type of incident	Clinical outcome
[13]	mercaptopurine (A); mercaptamine (B)	A prescribed instead of B by GP	Infant initially presented with nephropathic cystinosis. After one month on the wrong medication, the infant developed pancytopenia, but made a full recovery.
[14]	hydromorphone (C); morphine (D)	C administered instead of D by nurse	The patient (an elderly man) was discharged and suffered a fatal respiratory arrest on his way home.
[15]	gentamicin (E); clindamycin (F)	E administered instead of F	Not specified, but labelled ‘low harm’.
[16]	cisatracurium (G); vecuronium (H)	G dispensed instead of H by pharmacy technician and was administered	The patient was a 30+1 week old neonate. The error was realized immediately, and no changes to vital signs were observed.

To date, very few studies have looked at the formal properties of generic names and their relation to LASA errors, and these concerned USANs, not INNs [17]. To our knowledge, this is the first study to contextualize the formal properties of INNs within the WHO naming guidelines, and the first to look at semantic properties, by exploring the underlying conceptual system that groups names according to their pharmacology. Since INNs are the global pharmaceutical nomenclature from which national nomenclature systems are derived, study of the formal characteristics of INNs is of real importance to those interested in medications management, LASA errors, and patient safety. Identification of the factors that should be considered in naming new medications is important for products coming to market.

### 1.1 International Nonproprietary Names

International Nonproprietary Names (INNs) constitute a nomenclature of over 8,000 generic names for pharmaceutical substances. Some examples are given in Table 2. They are designated by the World Health Organization (WHO) and formally placed in the public domain to promote consistency in global communication between manufacturers, clinicians, prescribers, and patients. INNs are published in the six official languages of the United Nations and Latin, and are used by default as generic names in major national and regional pharmacopoeias, such as the British Pharmacopoeia and the European Pharmacopoeia [18]. Given their international status, the name designation process in place must encompass a wider conceptual system than that of regional naming councils, and naming guidelines must be robust and applied stringently.

**Table 2:** Examples of International Nonproprietary Names (INNs).

Year recommended	INN	Examples of current therapeutic indication(s) [19]
1955	chloramphenicol	Topical treatment of acute bacterial conjunctivitis
1965	betamethasone	Topical treatment of various dermatoses, including atopic dermatitis, psoriasis, and discoid lupus erythematosus
1975	levonorgestrel	72-hour emergency contraception
1985	mifepristone	Medical termination of developing intra-uterine pregnancy
1995	atorvastatin	Treatment of hypercholesterolaemia and prevention of cardiovascular disease
2005	golimumab	Treatment of rheumatoid arthritis, psoriatic arthritis, ankylosing spondylitis, and ulcerative colitis

INNs are designated in accordance with a set of naming guidelines, which give guidance on formal properties, such as spelling, phonology, hyphenation, and word length, and semantic properties, such as the use of stems to indicate pharmacological relationships between substances. Here we present an analysis of the formal and semantic properties of INNs, based on the naming guidelines of the WHO INN program, and discuss its clinical significance.

**Fig 1:**
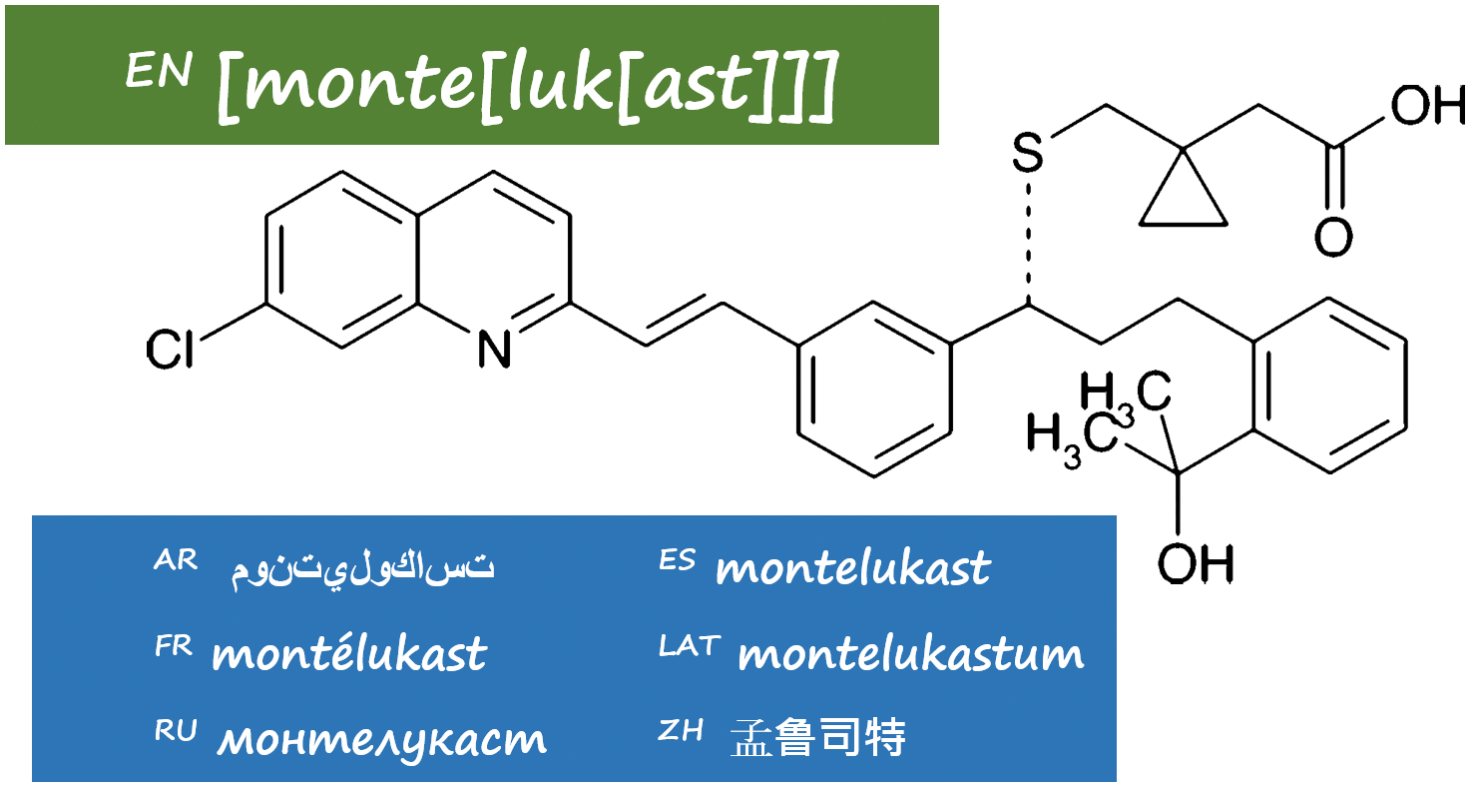
Montelukast in translated forms.

INNs are designated and promoted for international use and are restricted by a set of guidelines (‘principles’). Principles 1 and 2 are marked as ‘guiding principles’, and the WHO stipulates that “these primary principles are to be implemented by using the following secondary principles” [20]. The WHO naming principles in designating INNs that we explore here are provided in Table 3, and the full list of principles in S1 Table: *WHO naming principles for designation of INNs*. The other five principles were not looked at, since they pertained to particular classes (such as acids and salts), the regulation of INN designation, or purely phonetic aspects. Motivation behind these selected principles falls into four categories:

1. Usability: how easily can the name be used in the four modalities of language: reading, writing, listening, and speaking? Is the name memorable, and can it be printed on packaging?
2. Taxonomy: does the name indicate its position in the conceptual system, and interrelationships? This is a two-fold condition: there must be a robust and consistent conceptual system, and the formal properties of the names should be exploited to map on to the underlying conceptual system.
3. Clarity: how liable is the name to be confused with other names, both in the same system and in other systems?
4. International use: does the name adhere to the phonotactics of all languages in which it is used, and can it be easily transliterated into other languages with different alphabets or writing systems? (As illustrated by montelukast in Fig 1, INNs are published in Latin, English, Spanish, French, Russian, Chinese, and Arabic.).

**Table 3:** Selected WHO naming principles for designation of INNs (taken from [20]; sub-categorized here using square brackets).

Principle 1	International Nonproprietary Names (INNs) should be distinctive in [a] sound and [b] spelling. [c] They should not be inconveniently long and [d] should not be liable to confusion with names in common use.
Principle 2	[a] The INN for a substance belonging to a group of pharmacologically related substances should, where appropriate, show this relationship. [b] Names that are likely to convey to a patient an anatomical, physiological, pathological, or therapeutic suggestion should be avoided.
Principle 6	The use of an isolated letter or number should be avoided; hyphenated construction is also undesirable.
Principle 7	To facilitate the translation and pronunciation of INNs, "f" should be used instead of "ph", "t" instead of "th", "e" instead of "ae" or "oe", and "i" instead of "y"; the use of the letters "h" and "k" should be avoided. When devising an INN it is important to be aware of possible language problems. Since the name is used worldwide, not only should certain letters be avoided, but experts need to be aware of unsuitable connotations in the major languages spoken in the world.

Here we address the overarching research question: In relation to the WHO principles of INN designation, are there any threats to interpretation or translation in the form of:

1. Isolated numbers, isolated characters, or hyphens present in INNs (principle 6)
2. Prohibited graphs and digraphs present in INNs (principle 7)
3. Word length statistics (principle 1c)
4. Use of stems to indicate pharmacological relationships (principle 2a)
5. Patterns of similarity between INNs (principle 1d)

## Methods

The present analysis is concerned with the formal and semantic properties of International Nonproprietary Names, and was undertaken within the framework of naming guidelines (‘principles’) set out by the WHO [20,21]. In linguistics, the descriptors *formal* and *semantic* are often dichotomized to compare, respectively, the written or phonetic form of a word and its underlying conceptual meaning(s). These are inseparable facets of natural language, but the distinction is useful for analytical purposes [22].

**Fig 2:**
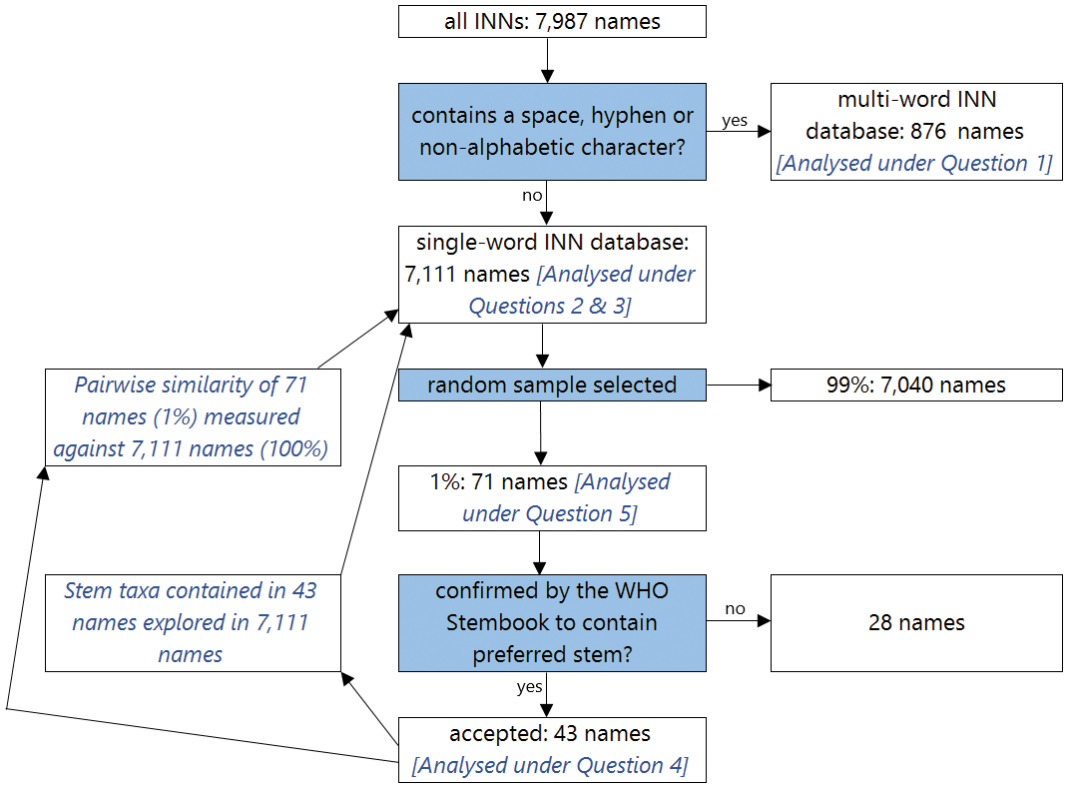
The sampling process.

As a starting point for the analysis, all INNs (n = 7,987) published in Recommended Lists from 1952 (when the INN program began) to August 2012 were digitized into an Excel spreadsheet. They were cross-verified on WHO MedNet. Two Excel databases were created, the first containing all single-word INNs (n = 7,111) and the second containing multi-word INNs (n = 876). The multi-word database was used for analysis under Question 1 concerning isolated numbers, characters, or hyphens. Any names containing a space or a non-alphanumeric character (such as a hyphen) were included in the multi-word database. The single-word database was used for analysis of Questions 2–5. Fig 2 summarizes the sampling process.

The use of stems (Question 4) was explored qualitatively in a randomly selected 1% segment (using the *RAND* function in Excel) of the single-word database (n = 71), as it was decided that for this question depth of analysis was preferable over breadth. The WHO Stembook [20] was used to verify that each INN in the 1% segment of the single-word database (n = 71) incorporated the correct stem(s). The 1% random sample was used as a starting point for our analysis, which explored the complete stem taxa of each stem and sub-stem contained in the 43 names.

For Question 5, concerning patterns of similarity between INNs, pairwise similarity was measured between the 1% random sample (71 INNs) against the entire single-word database (7,111 INNs). This was to limit processing power requirements, as measuring pairwise similarity between every name would result in over 50 million calculations, rather than 504,881 using a 1% sample, and manual inspection was needed for each pair to ascertain stem or sub-stem commonalities. The measurement of similarity used was Levenshtein edit distance (LED), which accounts for differences in word length [23] and is used in spell checking and predictive text software. It computes the number of insertions, deletions, or substitutions necessary to transform one string into another. For example, to transform *book* into *back*, *o* would be substituted for *a*, and the next *o* would be substituted for *c*, and the LED is 2. The LED is sensitive to differences in word length; for example, to transform mercaptopurine into mercaptamine, *a* replaces *o*, *m* replaces *p*, and *u* and *r* are deleted, so the LED is 4. Since the function’s algorithm is processed linearly through the characters in each string, matching sequences will decrease the LED.

For ease of reference, this paper will refer to hyperonyms as stems and hyponyms as sub-stems. Stems are underlined for brevity.

## Results

### 1. Are isolated numbers, isolated characters, or hyphens present in INNs? (principle 6)

The presence of isolated characters in an INN can endanger its interpretation. In word processed documents, the name may be split over two lines and the isolated character may be misinterpreted as a page number or footnote marker. In handwriting, a single letter is more easily misinterpreted than a word, because the reader cannot rely on other characters for context. Isolated numbers may be mistaken for part of the dosage instructions and result in wrong dosing.

In the multiword INNs database, seven INNs contained a hyphen, six of which hyphenate a Greek letter and alphanumeric code, e.g. peginterferon lambda-1a. There are no instances of a single isolated number or character, although INNs with a second alphanumeric word, such as ioflubenzamide ^131^I, create a risk of misinterpretation due to similarity between an upper-case eye, a lower case el, and the number one, even when printed, which all look the same in, for example, the Bauhaus 93 and Gill Sans MT fonts [24].

### 2. Are prohibited graphs and digraphs present in INNs? (principle 7)

Principle 7 is in place to facilitate the translation and correct pronunciation of INNs. By prohibiting graphs and digraphs such as <h> and <th>, which correspond to phonemes not predictably used in other languages, the principle facilitates translation of the name from Latin into the six official languages of the WHO (English, Spanish, French, Russian, Chinese, and Arabic), from which generic names in other languages are derived. There are wide variations in the pronunciation and writing systems in the world’s languages, so a simplification of English phonology is necessary. Principle 7 also serves Principle 1a and 1b (names should be distinctive in sound and spelling), by promoting the use of primary graphs with a one-to-one correspondence with phonemes, such as <f> (not <ph>) for phoneme /f/, and <e> (not <ae> or <oe>) for /e/. In this way, redundancy is avoided by using a single grapheme for each phoneme, and name length is reduced by one letter. INNs are therefore required to have an internal shallow orthography, i.e. one in which the correspondences between graphemes and phonemes are close to one-to-one.

Principle 2, which is given priority by the WHO, stipulates that names must show pharmacological relationship, and thus there is a conflict between principles 2 and 7. Although <ph>, <th>, <oe>, <y>, <h>, and <k> are prohibited, these (di)graphs are present in the list of stems used to form INNs (e.g. -kacin, -methasone, -orphinol) and so inevitably will be used. Furthermore, the Greek letter names *theta* and *kappa* are used to distinguish between similar preparations, for example, the biosimilars epoetin alpha, beta, theta, and zeta. Given the lower priority of Principle 7, it must be assumed that it should be adhered to unless the prohibited (di)graph forms part of a recommended stem, such as in amikacin. The numbers of instances of prohibited (di)graphs in INNs and stems are given in Table 4.

**Table 4:** Instances of prohibited (di)graphs in INNs and stems.

	ph	th	ae	oe	y	h	k	Total
All INNs containing the (di)graph	157	257	1	8	577	561	106	1,677
Stems containing the (di)graph	4	1	0	1	4	5	14	29

Many instances of ‘h’ are attributable to those in <ph> and <th>, although it does appear a further 171 times in the single-word INN database, either as an initial letter (e.g. hydrocortisone) or with ‘chlor’ (e.g. chlorpromazine). In total, the prohibited (di)graphs occurred 1,677 times in 1,036 INNs. Some INNs contained more than one, such as phthalylsulfathiazole (<ph>, <th>, <th>).

**Fig 3:**
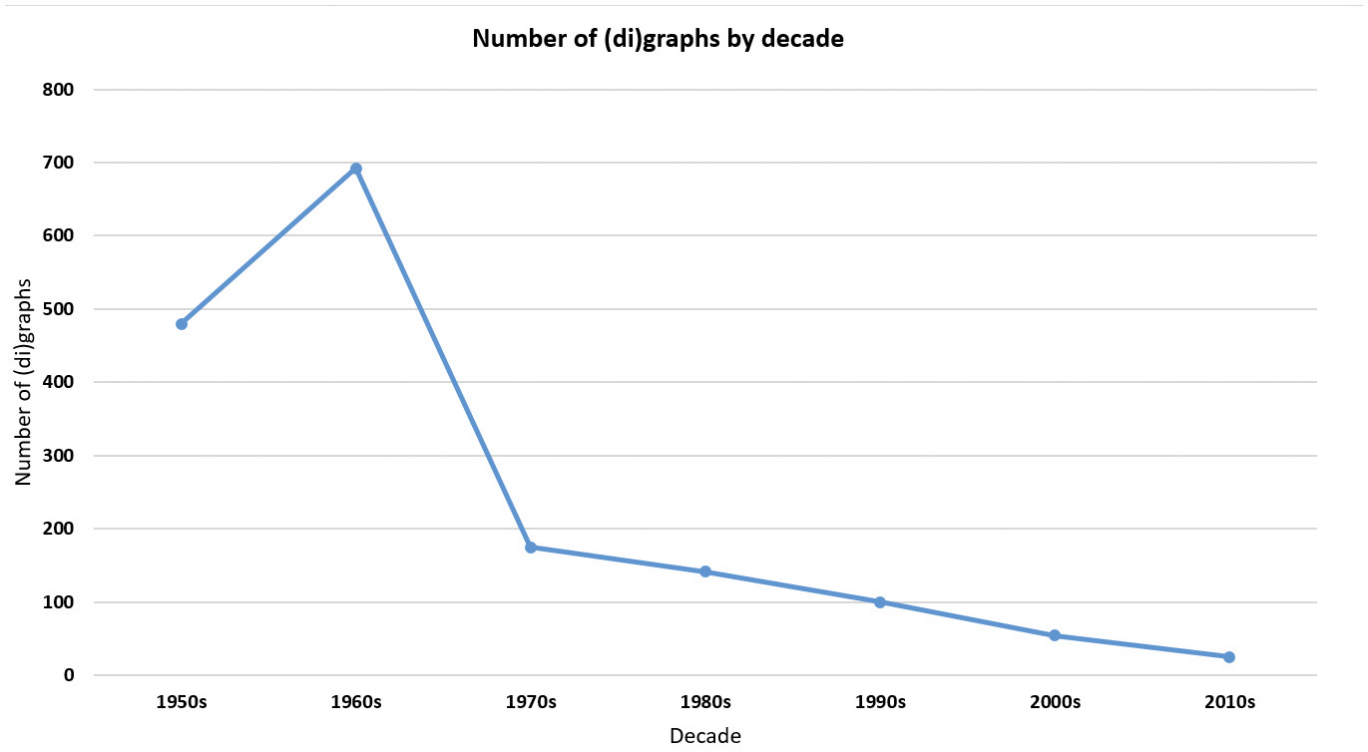
Number of prohibited (di)graphs contained in INNs published, by decade.

As shown by Fig 3 below, the majority of words containing prohibited (di)graphs were designated in the early stages of the INN programme, and few words continue to be designated. For example, although -methasone is the recommended stem, recently designated INNs have used the stem -metasone (e.g. dexamethasone in 1962, betamethasone in 1965, beclometasone in 1970 and alclometasone in 1979).

### 3. Word length statistics (principle 1c)

**Fig 4:**
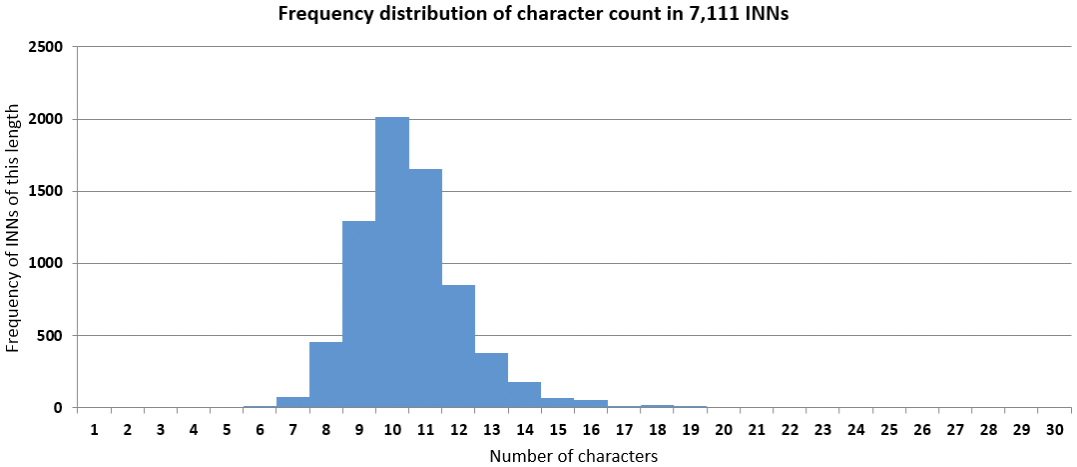
Mean character count in 7,111 INNs.

Fig 4 shows the character count distribution in single-word INNs. The mean number of characters across the entire dataset was 10.54, with a standard deviation of 1.73 characters. Both median and mode were 10, and the interquartile range was 9 to 11.

There are nine outliers with more than 20 characters, and these were all recommended before 1962. All nine contain at least one of the prohibited (di)graphs, which partly explains their unusual length, and at least seven syllables: sulfachlorpyridazine (20 letters), succinylsulfathiazole (21), methyldihydromorphine (21), phthalylsulfathiazole (21), ethylmethylthiambutene (22), phthalylsulfamethizole (22), sulfamethoxypyridazine (22), diiodohydroxyquinoline (22), and phenoxymethylpenicillin (23). It is notable that these names more closely resemble chemical names than do other INNs.

**Fig 5:**
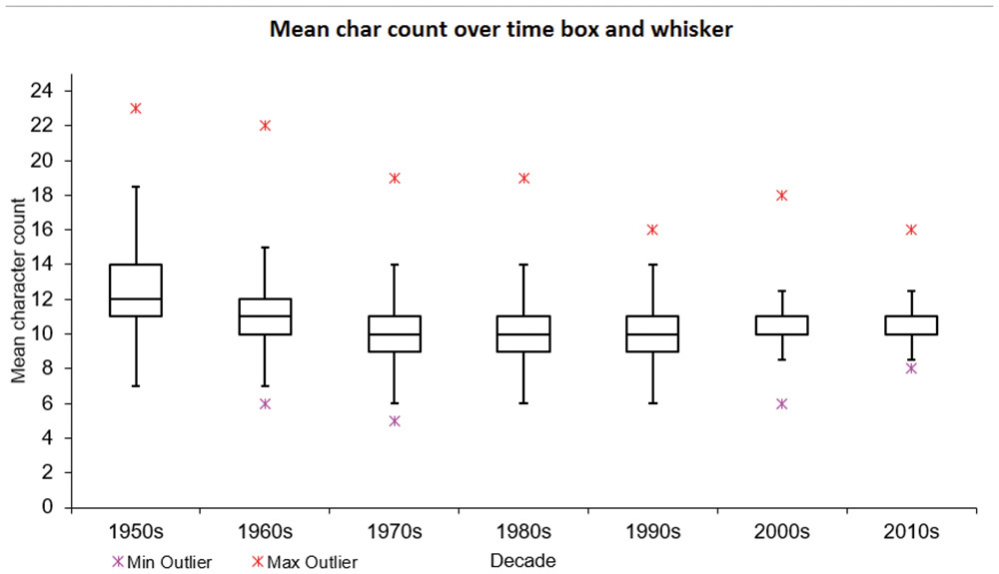
Mean character count of newly designated INNs by decade (This figure shows date of publication: medications may have been in prior use, but not published as INNs.).

As shown in Fig 5, the average length of INNs dropped sharply in the 1970s, and has remained steady since that time. The sudden drop coincided with the decreased use of (di)graphs prohibited under principle 7 from the 11^th^ list onwards.

### 4. Are stems used to indicate pharmacological relationships? (principle 2a)

Pharmacological relationships between substances are demonstrated by the use of a common stem [25] attached to a prefix, infix, suffix, or ‘freefix’. The use of stems and sub-stems creates a taxonomic conceptual system for INNs, and allows users to exploit this systematicity to improve retention, pronunciation, and recognition of the names. For example, montelukast comprises the stem -ast, and the sub-stem–lukast. Users can recognize montelukast as a medication used to treat asthma, or more specifically, a leukotriene receptor antagonist, and they can recognize that other names ending in–lukast have similar pharmacological actions to montelukast.

Stems and sub-stems may be suffixes (at the end of the name, such as granisetron and palonosetron), prefixes (at the beginning, such as artemether and arterolane), infixes (in the middle, such as mifepristone and ulipristal) and freefixes (which can appear anywhere, such as nabilone and nonabine). We encountered various types of taxonomy, which are outlined below. INNs analysed in the 1% sample are given in S2 Table: *Stems in 1% sample analysed semantically*. Of the 71 INNs, 43 were listed in the WHO Stembook as “names in which the preferred stem has been used in accordance with its definition”, and these were selected for analysis. The remaining 28 were either missing from the Stembook, or listed as not containing the preferred stem. For example, clortermine was listed under anorexics, which have the stem -orex, as an INN in which the preferred stem has not been used. By extending this finding to the entire sample it may be inferred that up to 40%, or 3,150 names, do not contain the preferred or correct stem.

In the 43 INNs analysed semantically, 20 (47%) contained ‘lone’ stems, which have no taxonomic relation to any other stems or sub-stems, such as -astine in clemastine, -azepide in pranazepide, and -cromil in nedocromil. Others contained a stem belonging to a larger taxon, such as montelukast in Table 5 (-ast: antiasthmatics or antiallergics, not acting primarily as antihistaminics > -lukast: leukotriene receptor antagonist). The stem taxon contains a hyperonym (-ast), and multiple hyponyms or sub-stems (-lukast, -trodast).

**Table 5:** —ast stem taxon.

sub-stem (hyponym)	stem (hyperonym)	Mode of action	Example INN	Therapeutic indication
(without sub-stem)	-ast	antiasthmatics or antiallergics, not acting primarily as antihistaminics	zaprinast	A selective PDE inhibitor, precursor to PDE-5 inhibitors such as sildenafil (Viagra) [26]
-lukast		leukotriene receptor antagonists	montelukast	Treatment for reversible bronchoconstriction [19]
-milast		phosphodiesterase type IV (PDE IV) inhibitors	cilomilast	Treatment for chronic obstructive airways disease and psoriasis [19]
-trodast		thromboxane A-2 receptor antagonists, antiasthmatics	seratrodast	Long-term management of asthma [27]
-zolast		leukotriene biosynthesis inhibitors	quazolast	Mediator release inhibitor [28]

An archetypal taxonomic system entails a clear tree hierarchy of concepts, based on hyperonyms and branching out to hyponyms. In the -ast taxon, and in many other INN taxa, the stem is a suffix, attaching to the end of the name, and its sub-stem is formed by adding an infix. This right-to-left display of taxonomy in montelukast is a predictable approach for the user, as they can first categorize the substance under its main stem, antiasthmatics (-ast), and then sub-categorize it as a leukotriene receptor antagonist (-lukast).

The sample of 43 names also contained three names for monoclonal antibodies, formed by the stem–mab (inolimomab, siplizumab and volociximab). As a newer branch of biochemistry, they adhere to a much stricter nomenclature and a more systematic approach.

With the exception of the first medication in this class, muromonab-CD3, names for monoclonal antibodies comprise a random prefix, followed by two infixes and a stem referring in a specified order to (a) the target class or disease class; (b) the source class on which the immunoglobulin sequence is based; and (c) the hyperonym -mab [19]. There are currently eight infixes to denote the source class and nine infixes to denote the target class. These may be combined freely with each other but the order in which they appear in the word is fixed. As an example, trastuzumab, which is a humanized monoclonal antibody directed against human epidermal growth factor receptor type 2 (HER2), can be decomposed as tras-tu—zu—mab, in which -tu- indicates that it targets tumour cells, and -zu- indicates that it is humanized (derived from a non-human antibody, which is then engineered to be more homologous with human antibodies). This is not an example of a three-tiered taxonomy, but rather two mutually independent parameters of classification under a single hyperonym, as shown in Table 6.

**Table 6:** —mab stem taxon.

sub-stem A (target)	sub-stem B (source)	stem	Definition	Example INNs
		-mab	Monoclonal antibody	
	-a-		rat	Not yet designated
	-axo-		rat/mouse	catumaxomab
	-e-		hamster	Not yet designated
	-i-		primate	Not yet designated
	-o-		mouse	solitomab
	-u-		human	namilumab
	-xi-		chimeric	pagibaximab
	-xizu-		chimeric/humanized	otelixizumab
	-zu-		humanised	natalizumab
-b(a)-			bacterial	tefibazumab
-c(i)-			cardiovascular	volociximab
-f(u)-			fungal	Not yet designated
-k(i)-			interleukin	lebrikizumab
-l(i)-			immunomodulating	infliximab
-n(e)-			neural	atinumab
-s(o)-			bone	romosozumab
-tox(a)-			toxin	urtoxazumab

However, there is no simple rule governing the position of stems and sub-stems in INNs. There are many other ways in which stems are ordered in the name, because stems and sub-stems may be prefixes, suffixes, infixes, or freefixes. The antiviral taxon, with hyperonymic stem vir, is below in Table 7. Vir is a freefix, and can appear anywhere in the name. Sub-stems of vir, including -viroc and -ciclovir, show that vir may be used as either a suffix or an infix, and so it is difficult for a user to immediately categorize a name under its hyperonym as an antiviral. A user may mistakenly categorize maraviroc under the stem *-oc, which does not exist. Other pharmacologically unrelated medications that happen to include the word part vir, such as virginiamycin and viridofulvin, may be misinterpreted as antivirals. In two other names in the sample, the sub-stem is a suffix and thus the name cannot be immediately recognized by its stem (such as: micinicate, with infix stem -nic(o)-; balaglitazone, with freefix stem gli).

**Table 7:** —vir stem taxon.

Sub-stem	Stem	Mode of action	Example INN	Therapeutic indication
	vir	Antivirals	efavirenz	Antiretroviral combination therapy in treatment of HIV-1 [19]
-amivir		Neuraminidase inhibitors	zanamivir	Treatment of influenza [19]
-asvir		Antivirals, hepatitis C virus (HCV) NS5A inhibitors	daclatasvir	Combination therapy for chronic hepatitis C [19]
-buvir		RNA polymerase (NS5B) inhibitors	dasabuvir	Combination therapy for chronic hepatitis C [19]
-cavir		Carbocyclic nucleosides	abacavir	Antiretroviral combination therapy in treatment of HIV [19]
-ciclovir		Bicyclic heterocycle compounds	valaciclovir	Treatment of herpes zoster and ophthalmic zoster [19]
-fovir		Phosphonic acid derivatives	adefovir	Treatment of chronic hepatitis B [19]
-gosivir		Glucoside inhibitors	celgosivir	Potential treatment of dengue (DENV) virus [29]
-navir		HIV protease inhibitors	saquinavir	Treatment of HIV-1 infected adults [19]
-previr		Hepatitis Virus C (HVC) protease inhibitors	telaprevir	Treatment of genotype 1 chronic hepatitis C [19]
-virine		Non-nucleoside Reverse Transcriptase Inhibitors (NNRTI)	etravirine	Treatment of HIV-1 in antiretroviral-experienced adults and children over 6 [19]
-viroc		CCR5 (Chemokine CC motif receptor 5) receptor antagonists	maraviroc	Treatment of detectable CCR5-tropic HIV-1 [19]

In many cases the orthographic form, instead of being exploited to facilitate interpretation, actually obfuscates the semantics of the names. While pharmacological relationship must be shown by using a common stem, some stems are distinguishable by only a single letter, such as -fenin (diagnostic aids; (phenylcarbamoyl)methyl iminodiacetic acid derivatives, e.g. lidofenin and disofenin) and -fenine (analgesics, glafenine derivatives, e.g. florifenine and glafenine) and many sub-stems in the -mab taxon comprise a single letter (cf. Table 6). In other cases, variation in the spelling of a stem (allomorphy) does not indicate a change in meaning (such as indoprofen and diprofene, or setiptiline and noxiptiline).

The use of -ine as the ending of INNs (the most frequent ending, 1502 of 7,111 names in the database) can falsely suggest a pharmacological relationship: for example, riodipine in the sample contains the stem -dipine (calcium channel blockers, 1,4-dihydropyridine derivatives), but could be mistakenly interpreted under -pine (tricyclic compounds). Homophonic yet distinct stems can also be misleading, such as -micin (gentamicin and netilmicin) and -mycin (erythromycin and kanamycin), denoting antibacterials. This example also reveals inconsistencies in the semantic motivation of stems. Here, the stem distinguishes the genus from which it is derived, -micins from Micromonospora and -mycins from Streptomyces, and not the pharmacological group, thereby blurring the boundaries between meanings of names containing these stems.

As in the INNs for monoclonal antibodies, meaning may depend on the order of stem concatenation. For example, when -fos is used as a suffix, it is a hyperonym denoting “insecticides, anthelminthics, pesticides etc., phosphorus derivatives”, e.g. uredofos, but when it appears as an infix or a prefix, it is the hyponym (sub-stem) denoting “various pharmacological categories belonging to fos, other than those above”, e.g. benfosformin [19]. In other cases, position in the word does not indicate semantic difference, such as grel and vir, which have the same meaning regardless of whether they are used as an infix or a suffix.

### 5. Are there patterns of similarity between INNs? (principle 1d)

Of 504,881 total pairwise similarity measures, 1,463 had a Levenshtein edit distance of between 1 and 4, i.e. no more than 4 characters or deletions distinguished the names. 33% (478 pairs) of these shared a stem, and 88 (6%) also a sub-stem. These included prefixal stems such as arte- in arterolane-arteflene, with an LED of 3, and the prefixes salazo- and sulfa- present together in salazosulfadimidine-salazosulfamide, with an LED of 4. There were also pairs that had the same final letters but did not share a stem indicating pharmacological relationship, such as lagatide-giractide, in which the former has the stem -tide and the latter has the unrelated stem -actide. Similarity statistics are given in Table 8. The table indicates the strong influence of stems on similarity; the more similarity between two names, the more likely it is that they will share a stem and/or a sub-stem.

**Table 8:** Similarity statistics.

Levenshtein Edit Distance	Frequency	Examples	Percentage sharing a stem	Percentage sharing both a stem and a sub-stem
1	2	alverine-salverine; amezepine-mezepine	100%	0%
2	19	peplomycin-peliomycin; clortermine-clormercaine	84.2%	26.3%
3	240	inolimomab-solitomab; pelubiprofen-flurbiprofen	62.9%	13.3%
4	1202	pibutidine-sufotidine; meclofenoxate-metofenazate	25.7%	4.2%

The sample group contained four monoclonal antibody substances, represented by the -mab stem family (icrumab, inolimomab, siplizumab and volociximab). Of the pairs with an LED of <5, names for monoclonal antibodies occurred only in pairs, and did not display a high degree of similarity with names with other stems. Pairs of monoclonal antibodies presented a high proportion of all stem-based similarities (3 with LED = 2; 27 with LED = 3; 58 with LED = 4; total 88). For example, siplizumab scored an LED of 2 with both ruplizumab and teplizumab, as they are only distinguishable by the first two letters. They share the stem -mab, and both sub-stems -zu- and -li-. In words such as these, when seven letters are predetermined by the norms of the designation process, the random prefix is responsible for the essential role of distinguishing the name from its co-hyponyms.

## Discussion

The WHO naming principles considered in this paper have not been strictly observed. Only the first two are prioritized, and these relate to the fourth and fifth objectives in this paper. There is a clear trend towards stricter compliance with WHO principles after 1960, as most of the extemely long names (20 characters or more) or those with prohibited (di)graphs were designated in the 1950s. These earlier INNs are still in use, and it is difficult to amend names after publication in a recommended list in the public domain. However, we unearthed a more pervasive and important problem: the inherent tension between using common stems to indicate pharmacological meaning and minimizing similarity in nomenclature to reduce confusion (see 4.2).

Another problem is that not all the principles are delimited and quantifiable, meaning they are difficult to follow and almost impossible to regulate. For example, principle 1 (“International Nonproprietary Names (INNs) should be distinctive in sound and spelling. They should not be inconveniently long and should not be liable to confusion with names in common use”) [19] does not provide quantifiable criteria, such as a character limit, or a maximum degree of similarity.

### 4.1 Formal Properties of Medication Names

In general, medication name designation complied with the WHO principles relating to formal properties of nomenclature.

Some INNs contained hyphens, but no isolated letters and numbers were found. 1,677 INNs contain prohibited (di)graphs such as <ph> rather than <f>. Words without a one-to-one grapheme-phoneme correspondence, such as *thorough* (eight graphs, four phonemes–/θərə/), take longer to be recognized in reading. Words containing graphs (letters) without direct correspondence to their phonemes have been found to take longer to be recognized in reading [30]. For example, *fooph (3 phonemes and 5 graphs) will take longer to recognize than *fruls (5 phonemes and 5 graphs), because the reader first interprets the <p> in *fooph as the phoneme /p/, but then on meeting <h> is forced to reinterpret as the phoneme /f/. Recognition time is further increased when non-correspondences occur earlier in the word, as the reader would not be able to infer meaning from the context of earlier letters. When analysed into single graphs, the digraphs <ph>, <th>, <ae>, and <oe> do not have direct correspondence with the primary phonemes of each graph and thus may increase recognition time and reduce usability.

Word lengths of INNs are relatively stable diachronically, with an overall mean character count of 10.54, but there are a few INNs with more than 20 characters. Long INNs, such as phenoxymethylpenicillin (23 characters), are problematic to fit on packaging; the NPSA in the UK, for example, has recommended a minimum font size of 16 points for the generic name [31]. Long INNs risk being hyphenated and running to multiple lines when printed, reducing legibility and increasing the risk of misunderstanding or confusion with other names. Recognition time will be increased by high character count, low frequency of the words and perceived ‘nonwords’ [32,33], and this effect may be magnified by variation in the prescribing frequency of INNs [34].

### 4.2 Potential for LASA errors

We have identified a tension between WHO principles stipulating the use of stems to indicate pharmacological relationships and those aiming to reduce similarity in nomenclature.

If our numbers are representative of the totality of drug names, some soft inferences can be made. Regarding use of stems (question 4), 23 out of 43 names displayed no problems in form or taxonomy. The remaining 20 (47%) presented at least one problem, such as unpredictable ordering of stems and sub-stems, variation in spelling (allomorphy), homophony, and similar-looking but unrelated stems. By extension, we might estimate that 3,308 INNs in the total sample may be problematic. Secondly, 15 of 71 names (21%) are distinguishable from another name by only one or two letters (LED is < = 2). By extension, we might estimate that 1,502 INNs in the total sample may share this degree of similarity with at least one other name.

Although these numbers are conjectural, as the degree of overlap to the total target lexicon is uncertain, results from analysis of a 1% sample in fact pertain to a larger proportion of the entire sample. We compared the 1% sample of INNs against 100% for pairwise similarity and found that those sharing a stem are more likely to display a high degree of orthographic similarity, a finding that can be extended to the other 99% of INNs in the sample. Similarly, our analysis of the use of stems investigated over thirty stem taxa in depth and so is relevant to all names containing stems from those taxa in the entire sample.

#### 4.2.1 Stems indicating pharmacological relationships

There is no single way for a user to predict meaning from an INN, although we found consistency within some taxa, e.g. monoclonal antibodies and antivirals. In some cases, a single letter will be used to distinguish between unrelated pharmacological groups, whereas in other cases there is simply wide spelling variation that does not contribute to meaning. The burden on users is high: they need to understand the meanings of stems and the layout of the taxonomy, and also to learn when to ignore spelling variations and when to take note of a single letter distinguishing meaning. They must understand that meaning may be motivated at the supramorphemic level by the class of affix and the concatenation of stems and sub-stems. Consequently, most clinicians make little use of pharmacological nomenclature in routine practice, relying instead on the appearance and sounds of whole words, memorized during experiential learning in clinical environments, and on pharmaceutical company presentations, and preferring to use simpler brand names, mitigating against the use of generic names.

#### 4.2.2 Patterns of similarity between INNs

Pharmacologically related substances whose names show their relationship by the use of a common stem, and those that are unrelated but erroneously shared a stem, have a higher level of similarity and are thus more likely to be confused, such as:

1. arterolane-arteflene (arte-: antimalarial agents, artemisinin related compounds);
2. salazosulfadimidine-salazosulfamide (sal-: analgesic anti-inflammatories; salazo-: phenylazosalicyclic acid derivatives antibacterial) (sulfa-: anti-infectives, sulfonamides); and
3. siplizumab-teplizumab (-mab: monoclonal antibodies; -li- and -zu-: humanized, targeting the immune system)

This suggests that the use of the stem system may actually increase the risk of confusion and thus endanger patients. However, without it users of pharmaceutical names would need to learn the meanings of all medicines by rote, without the benefit of common affixes. Thus, two primary objectives of the WHO—usability and taxonomy—are in competition with each other, and this is compounded by a messy underlying taxonomy.

We found significant levels of similarity between pharmacologically related INNs. Hence, we have identified dissonance between sub-principles 1d (mitigating the risk of confusion) and 2a (stipulating the display of pharmacological relationship). These sub-principles compete in the pursuit of the primary goals, namely, reduction of the risk of confusion with other medication names and accurate perception of the meanings of INNs. This conflict can only be mitigated when the stem system is predictably structured, to avoid increasing the risk of confusion.

## Limitations

We have looked at formal and semantic properties of International Nonproprietary Names within a selection of the WHO naming principles. We have examined orthographic form, but not phonetic form, and used only one similarity measurement method. We analysed only INNs in English, but studies are underway adapting these methods to the analysis of translated forms (for example, in Latin, Spanish, French, Russian, Chinese, and Arabic), and evaluating their compliance with WHO naming principles [19]. Further work is needed to explore the clinical implications of this work.

A small sample (1%) was necessary, owing to available resources of time and processing power. We estimate that analysis of the complete corpus would require up to 1,500 person-hours. Our analysis was also restricted to names considered acceptable by the WHO, as it was beyond our scope to analyse drug names that do *not* conform to WHO guidelines.

## Conclusions

INNs carry out different functions depending on the user, and so they must be understandable at multiple levels. For patients and non-professionals they must be recognisable and pronounceable, and simultaneously their meaning and pharmacological relationships with other names must be understood by health professionals if medication errors are to be avoided.

The pharmaceutical nomenclature and its peripheral systems of nomenclature (such as pathology, anatomy, nosology, etc.) are extensive and complex. It is inevitable that a taxonomy developed over a number of decades will contain some broken links and general inconsistencies, but these should not work to the detriment of the overall aim of the system.

Like two sides of a coin, the formal and semantic aspects of language are inextricably linked, and it is impossible to speak of formal motivation without referring to semantic motivation. Formal aspects of INNs are motivated by the semantics they represent, and while the formal realisation of INNs is, at times, conducive to conveying their meaning, it can also misrepresent meaning and increase the risk of confusion.

Findings on word length (Question 3) were closely aligned with a similar study on USANs [17], and have highlighted certain (di)graphs that are prohibited but nevertheless still in use (Question 2). Results for Questions 4 and 5, regarding the use of stems and similarity, have exposed a tension in the INN nomenclature, and highlighted the need for further research into the exact interplay between these naming principles and their implementation.

## Supporting Information

S1 Table WHO naming principles for designation of INNs. (DOCX)

S2 Table Stems in 1% sample analysed semantically. (DOCX)
