# Supplementary material for: Patient Safety in Medication Nomenclature: Orthographic and Semantic Properties of International Nonproprietary Names
Source: PLoS One. 2015 Dec 23;10(12):e0145431. doi: 10.1371/journal.pone.0145431 (PMC4689353; doi:10.1371/journal.pone.0145431)
Supplement: S1 Table — (DOCX) [file pone.0145431.s001.docx]

# Table S1: WHO naming principles for designation of INNs

| Principle 1  (guiding principle) | International Nonproprietary Names (INN) should be distinctive in sound [a] and spelling [b]. They should not be inconveniently long [c] and should not be liable to confusion with names in common use [d]. |
| --- | --- |
| Principle 2  (guiding principle) | The INN for a substance belonging to a group of pharmacologically related substances should, where appropriate, show this relationship [a]. Names that are likely to convey to a patient an anatomical, physiological, pathological or therapeutic suggestion should be avoided [b]. |
| Principle 3 | In devising the INN of the first substance in a new pharmacological group, consideration should be given to the possibility of devising suitable INNs for related substances, belonging to the new group. |
| Principle 4 | In devising INNs for acids, one-word names are preferred; their salts should be named without modifying the acid name, e.g. "oxacillin" and "oxacillin sodium", "ibufenac" and "ibufenac sodium" |
| Principle 5 | INNs for substances which are used as salts should in general apply to the active base or the active acid. Names for different salts or esters of the same active substance should differ only in respect of the name of the inactive acid or the inactive base. |
| Principle 6 | The use of an isolated letter or number should be avoided; hyphenated construction is also undesirable. |
| Principle 7 | To facilitate the translation and pronunciation of INN, "f" should be used instead of "ph", "t" instead of "th", "e" instead of "ae" or "oe", and "i" instead of "y"; the use of the letters "h" and "k" should be avoided. When devising an INN it is important to be aware of possible language problems. Since the name is used worldwide, not only should certain letters be avoided, but experts need to be aware of unsuitable connotations in the major languages spoken in the world. |
| Principle 8 | Provided that the names suggested are in accordance with these principles, names proposed by the person discovering or first developing and marketing a pharmaceutical preparation, or names already officially in use in any country, should receive preferential consideration. |
| Principle 9 | Group relationship in INNs (see Guiding Principle 2) should if possible be shown by using a common stem. |
