# Supplementary material for: Patient Safety in Medication Nomenclature: Orthographic and Semantic Properties of International Nonproprietary Names
Source: PLoS One. 2015 Dec 23;10(12):e0145431. doi: 10.1371/journal.pone.0145431 (PMC4689353; doi:10.1371/journal.pone.0145431)
Supplement: S2 Table — (DOCX) [file pone.0145431.s002.docx]

# Table S2

**Stems in 1% sample analysed semantically (stems,** *sub-stems***)**

| **INN** | **Actions and uses** | **freefix** | **prefix** | **infix** | **suffix** |
| --- | --- | --- | --- | --- | --- |
| acridorex | Anorexic agent [1] |  |  |  | **‑orex** |
| alverine | Relieves smooth muscle spasm in irritable bowel syndrome and dysmenorrhoea [2] |  |  |  | **‑verine** |
| amezepine | Antidepressant [1] |  |  | *‑ze‑* | **‑pine** |
| arterolane | Antimalarial; also known as RBx11160 [3] |  | **arte‑** |  |  |
| balaglitazone | Peroxisome proliferator‑activated receptor (PPAR) gamma partial agonist, and potential treatment for diabetics [4] | **gli** |  |  | *‑tazone* |
| benfosformin | Antihyperglycaemic agent [1] |  |  | *‑fos‑* | **‑formin** |
| cebaracetam | Amide-type nootropic agent [5] |  |  |  | **‑racetam** |
| cipamfylline | Phosphodiesterase (type IV) inhibitor. Selective inhibitor of tumour necrosis factor alfa production [1] |  |  |  | **‑fylline** |
| clemastine | Allergic rhinitis and dermatoses [2] |  |  |  | **‑astine** |
| conivaptan | Vasopressin receptor antagonist. Treatment of hyponatraemia [2] |  |  |  | **‑vaptan** |
| disofenin | Imaging agent of liver and kidneys [1] |  |  |  | **‑fenin** |
| doxaprost | Bronchodilator [1] | **prost** |  |  |  |
| enolicam | Cyclooxygenase and lipoxygenase inhibitor. Anti-inflammatory, antirheumatic, antiarthritic agent [1] |  |  |  | **‑icam** |
| fluperlapine | Antidepressant, neuroleptic agent. Never marketed [1] |  |  | *‑a‑* | **‑pine** |
| indanidine | Partial alpha1-adrenoceptor agonist, alpha2‑adrenoceptor antagonist; antihypertensive agent [1] |  |  |  | **‑nidine** |
| indoprofen | Analgesic. Nonsteroidal anti-inflammatory agent [1] |  |  |  | **‑profen** |
| inolimomab | Murine monoclonal antibody targeting interleukin-2 receptor-alpha chains [6] |  |  | *‑lim‑  ‑o‑* | **‑mab** |
| ioxilan | Radiographic contract medium for urography or angiography [2] |  | **io‑** |  |  |
| lagatide | Antidiarrhoeal [5] |  |  |  | **‑tide** |
| levopropylhexedrine | CNS stimulant; nasal decongestant; vasoconstrictor. Used as an anorectic in Germany. [1] |  |  |  | **‑drine** |
| meprylcaine | Local anaesthetic used in dentistry [7] |  |  |  | **‑caine** |
| micinicate | Vasodilator, spasmolytic agent [1] |  | **nico‑** |  | *‑nicate* |
| montelukast | Treatment and prophylaxis of asthma symptoms [2] |  |  | *‑luk‑* | **‑ast** |
| nedocromil | Management of mild to moderate bronchial asthma; allergic conjunctivitis [2] |  |  |  | **‑cromil** |
| nonabine | Cannabis analogue. Antiemetic agent [1] | **nab** |  |  |  |
| palonosetron | 5HT_3_ receptor antagonist. Antiemetic used in cancer chemotherapy [2] |  |  |  | **‑setron** |
| pelubiprofen | Analgesic. Non-steroidal anti-inflammatory drug [8] |  |  |  | **‑profen** |
| peplomycin | Antineoplastic antibiotic [1] |  |  |  | **‑mycin** |
| phenylephrine | Relief of nasal congestion associated with colds and hayfever [2] |  |  |  | **‑frine** |
| pibutidine | Histamine‑H2‑receptor antagonist [5] |  |  |  | **‑tidine** |
| pimelautide | Immunostimulant [1] |  |  |  | **‑tide** |
| pranazepide | Allergic conjunctivitis [2] |  |  |  | **‑azepide** |
| riodipine | Calcium channel blocker [5] |  |  |  | **‑dipine** |
| salazosulfadimidine | Antibacterial agent [1] |  | **sal‑  sulfa‑** | *‑azo‑* |  |
| setiptiline | Tetracyclic antidepressant [9] |  |  |  | **‑tiline** |
| siplizumab | Humanized monoclonal antibody targeting CD2 receptors on T cells and natural killer cells [10] |  |  | *‑li‑  ‑zu‑* | **‑mab** |
| sulfamerazine | Sulfonamide. Cream‑coloured powder, darkens on exposure to light [1] |  | **sulfa‑** |  |  |
| tilmacoxib | COX-2 inhibitor [11] |  |  |  | **‑coxib** |
| trifenagrel | Inhibitor of arachidonate and collagen-induced aggregation of platelets [12] | **grel** |  |  |  |
| ubenimex | Competitive protease inhibitor [13] | **imex** |  |  |  |
| ulipristal | Emergency contraception within 120 hours [2] |  |  | **‑pris‑** |  |
| valaciclovir | Treatment of herpes zoster and ophthalmic zoster [2] | **vir** |  | *‑ciclo‑* |  |
| volociximab | Chimeric monoclonal antibody targeting human α5β1 integrin [14] |  |  | *‑ci‑  ‑xi‑* | **‑mab** |

## References

1. Ganellin, CR, Triggle, DJ. Dictionary of Pharmacological Agents. London: Chapman & Hall; 1996.
2. Electronic Medicines Compedium, eMC. Home page [cited 22 Jul 2015]. Available from: <http://www.medicines.org.uk/emc/>.
3. Uhlemann, A-C, Wittlin, S, Hugues, M, Bustamente, LY, Krishna, S. Mechanism of Antimalarial Action of the Synthetic Trioxolane RBX11160 (OZ277). Antimicrob Agents Chemother. 2007;51(2):667–672.
4. Youssef, JA, Badr, MZ. Peroxisome Proliferator-Activated Receptors. New York: Humana Press; 2013.
5. World Health Organization, WHO. WHO Stembook 2009: The use of stems in the selection of International Nonproprietary Names (INN) for pharmaceutical substances. 2009 [cited 22 Jul 2015]. Available from: http://www.who.int/medicines/services/inn/StemBook2009.pdf
6. Bay, JO, Dhédin, N, Goerner, M, Vannier, JP, Marie-Cardine, A, Stamatoullas, A, et al. Inolimomab in steroid-refractory acute graft-versus-host disease following allogeneic hematopoietic stem cell transplantation: retrospective analysis and comparison with other interleukin-2 receptor antibodies. Clin Transplant. 2005;80(6):782–788.
7. Morton, I, Morton, I, Hall, JM. Concise Dictionary of Pharmacological Agents. The Netherlands: Kluwer Academic Publishers; 1999.
8. Nuutinen, L, Raj, PP. An overview of current and investigational non-narcotic drugs for treatment of acute and chronic pain. Curr Rev Pain. 1998;2(3):187–192.
9. Miyamoto, S. Setiptiline Maleate. In: Stolerman, IP, editor. Encyclopedia of Psychopharmacology. Springer: Berlin; 2010.
10. Langley, RG, Papp, K, Bissonnette, R, Toth, D, Matheson, R, Hultquist, M, et al. Safety profile of intravenous and subcutaneous siplizumab, an anti-CD2 monoclonal antibody, for the treatment of plaque psoriasis: results of two randomized, double-blind, placebo-controlled studies. Int J Dermatol. 2010;49(7):818–828.
11. Yamamoto, H, Kondo, M, Nakamori, S, Nagano, H, Wakasa, KI, Sugita, Y, et al. JTE-522, a cyclooxygenase-2 inhibitor, is an effective chemopreventive agent against rat experimental liver fibrosis. Gastroenterology 2003;125(2):556–571.
12. Mukhopadhyay, C, Tapaswi, PK, Drew, MGB. Room temperature synthesis of tri-, tetrasubstituted imidazoles and bis-analogues by mercaptopropylsilica (MPS) in aqueous methanol: application to the synthesis of the drug trifenagrel. Tetrahedron Lett. 2010;51(30):3944–3950.
13. Sekine, K, Fujii, H, Abe, F. Induction of apoptosis by Bestatin (ubenimex) in human leukemic cell lines. Leukemia. 1999;13(5):729–34.
14. Bell-McGuinn, KM, Matthews, CM, Ho, SN, Barve, M, Gilbert, L, Penson, RT, et al. A phase II, single-arm study of the anti-α5β1 integrin antibody volociximab as monotherapy in patients with platinum-resistant advanced epithelial ovarian or primary peritoneal cancer. Gynecol Oncol. 2011;121(2):273–279.
